# IFN‐γ promotes radioresistant Nestin‐expressing progenitor regeneration in the developing cerebellum by augmenting Shh ligand production

**DOI:** 10.1111/cns.14485

**Published:** 2023-10-03

**Authors:** Jian Hu, Zixuan Wang, Biao Gong, Liyuan Feng, Yan Song, Shuo Zhang, Lin Wang, Yanghui Qu, Gen Li, Li Zhang, Chaonan Zheng, Fang Du, Peng Li, Yuan Wang

**Affiliations:** ^1^ Pediatric Cancer Center, Jiangsu Key Laboratory of Neuropsychiatric Diseases, Department of Pharmacology, College of Pharmaceutical Sciences Soochow University Suzhou China; ^2^ Department of Pharmacognosy and Traditional Chinese Pharmacology, College of Pharmacy Army Medical University Chongqing China

**Keywords:** cerebellum, IFN‐γ, irradiation, NEP regeneration, Shh ligand

## Abstract

**Background:**

Patients with brain tumors, especially pediatric brain tumors such as cerebellar medulloblastoma, always suffer from the severe side effects of radiotherapy. Regeneration of neural cells in irradiation‐induced cerebellar injury has been reported, but the underlying mechanism remains elusive.

**Methods:**

We established an irradiation‐induced developing cerebellum injury model in neonatal mice. Microarray, KEGG analysis and semi in vivo slice culture were performed for mechanistic study.

**Results:**

Nestin‐expressing progenitors (NEPs) but not granule neuron precursors (GNPs) were resistant to irradiation and able to regenerate after irradiation. NEPs underwent less apoptosis but similar DNA damage following irradiation compared with GNPs. Subsequently, they started to proliferate and contributed to granule neurons regeneration dependent on the sonic hedgehog (Shh) pathway. In addition, irradiation increased Shh ligand provided by Purkinje cells. And microglia accumulated in the irradiated cerebellum producing more IFN‐γ, which augmented Shh ligand production to promote NEP proliferation.

**Conclusions:**

NEP was radioresistant and regenerative. IFN‐γ was increased post irradiation to upregulate Shh ligand, contributing to NEP regeneration. Our study provides insight into the mechanisms of neural cell regeneration in irradiation injury of the developing cerebellum and will help to develop new therapeutic targets for minimizing the side effects of radiotherapy for brain tumors.

## Graphical abstract

NEP was radioresistant and regenerative in the irradiation‐injured cerebellum. IFN‐γ derived from microglia was increased post irradiation to upregulate Shh ligand expression by Purkinje cells, which contributed to NEP proliferation.

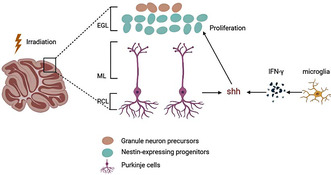

## 1 INTRODUCTION

Radiotherapy has become one of the main treatment methods for primary and metastatic brain tumors because their complex anatomical structure and the existence of the blood–brain barrier make it difficult to effectively control by surgery and drug treatment.^1^ However, radiotherapy inevitably causes irradiation brain injury. The survival and function of normal neural cells are seriously affected, and sequelae such as cognitive impairment, ataxia, and endocrine dysfunction occur. The incidence of irradiation brain injury after radiotherapy for nasopharyngeal carcinoma was 1.9% ~ 5%,^2^ that for low differentiated glioma and brain metastases was 1% ~ 24%^3^ and 8% ~ 20%,^4^, ^5^ respectively, while that for pediatric brain tumors, such as cerebellar medulloblastoma, is even higher, more than 60% of radiotherapy patients, especially young children, will have severe radiotherapy sequelae due to the damaged cerebellum.^6^ These side effects affect the quality of life of patients for a long time, and unfortunately, there is currently a lack of effective treatment or strategies to alleviate the sequelae of this radiotherapy. In our current study, we will utilize a model of cerebellar irradiation injury in neonatal mice to mimic the radiotherapy‐induced cerebellum dysfunction for mechanistic study.

The total volume of the cerebellum accounts for approximately 10% of the entire brain, yet it contains more than half of the total number of neurons in the whole brain. The cerebellum controls the balance and movement of the body and assists in the development of cognitive functions.^7^ The development of the cerebellum has a unique pattern, mainly in infancy, and has strict stratification; from the outside to the inside, the cerebellum is divided into an external granule layer (EGL) composed of Math1‐expressing granule neuron precursors (GNPs), a molecular layer (ML), a Purkinje cell layer (PCL), an internal granule layer (IGL) composed of mature granule neurons (GNs) and the innermost white matter (WM).^8^, ^9^ GNs are the most abundant and dominantly functional neurons in the cerebellum and are proliferated and differentiated by GNPs in late embryonic development and the early postnatal period under the control of the sonic hedgehog (Shh) signaling pathway.^10^, ^11^ In mice, GNPs are located in the EGL during the first 3 weeks of life, and they sense the Shh ligand secreted by Purkinje cells, which activate the Shh pathway to promote GNP proliferation. GNPs proliferate while continuously migrating inward into the IGL and gradually differentiate into mature GNs.^12^, ^13^, ^14^ After 21 days, all GNPs migrate to the IGL to complete differentiation, so the EGL disappears and the cerebellum is fully developed. Therefore, GNP proliferation and differentiation are essential for the normal development and functional formation of the cerebellum. GNPs are highly vulnerable to endogenous damage such as genetic mutations and exogenous damage, including irradiation. Studies have shown that a 2 Gy dose of irradiation can damage 70% of the cells in the cerebellum EGL of newborn rats.^15^ Studies have also shown that EGL can be reconstructed in a relatively short period of time and eventually form a normal adult cerebellum.^16^ However, how irradiation‐damaged cells in the cerebellum regenerate and the mechanisms involved in the process have not been fully studied.

Nestin is a type VI intermediate filament protein^17^ that is generally expressed in neural stem cells and glial cells and is mainly distributed in the cytoplasm and cell fibers.^18^ Recent studies have also shown that Nestin is expressed in progenitor cells of various lineages.^19^, ^20^ In our previous studies, we identified a population of Nestin‐expressing progenitors (NEPs) located in the deep part of the EGL. Unlike GNPs, NEPs present a quiescent status in the developing cerebellum of normal mice, but they are more tumorigenic in Shh‐type medulloblastoma than GNPs based on Shh antagonizing receptor *Pathed1* (*Ptc1*) gene deficiency. However, although the residence and gene expression profile are distinct in NEPs and GNPs, NEPs are neuronal lineage commitment cells that develop into mature GNs both in vitro and in vivo. Moreover, NEPs share the same ability to respond to the Shh ligand, activating Shh signaling to promote proliferation.^21^ More recently, NEPs showed reprogramming plasticity in neural cell differentiation.^22^ The properties of NEPs lead us to speculate that they might be resistant to radiotherapy‐induced damage and regenerate in the radiotherapy‐injured cerebellum. Therefore, we established a radiotherapy‐induced cerebellar injury model in neonatal mice to identify the capacity of NEPs in the postinjury regeneration and investigate the underlying mechanisms.

## 2 MATERIALS AND METHODS

### 2.1 Animals

*Math1‐GFP* mice, *Nestin‐CFP* mice, and *Nestin‐CreER* ^*T2*^ */R26R‐GFP* mice have been used and described previously.^21^ *Shh‐Cre‐GFP* mice were kindly provided by Dr. Jian‐quan Chen Soochow University. INF‐γ^null/null^ mice were kindly provided by Dr. Zeng‐jie Yang at Fox Chase Cancer Center. Wild‐type (WT) *C57BL/6* mice were purchased from Beijing Vital River Laboratory Animal Technology Co., Ltd. All animals were maintained in the SPF animal facility of Soochow University and Chongqing Army Medical University.

### 2.2 Irradiation

Postnatal Day 4 (P4) mouse pups were irradiated with a single dose of 4 Gy. The pups' bodies were covered by tinplate with head exposure. After irradiation, the cerebella of irradiated and nonirradiated control pups were collected for sectioning, quantitative PCR, and Western blotting assays. For in vitro cell irradiation, GNP and NEP cells were isolated and purified from the cerebella of naïve P4 *Math1‐GFP* and *Nestin‐CFP* mice, respectively, and cells were plated on PDL‐coated coverslips or 24‐well plates and then irradiated at 2 Gy. After irradiation, cell numbers were counted, and immunofluorescence and comet assays were performed.

### 2.3 Cell preparation

For GNP and NEP cell isolation, we followed a previously described protocol.^21^ Briefly, cerebella were harvested from *Math1‐GFP* or *Nestin‐CFP* mice at P4. Dissected EGL was digested in a solution containing 10 U/mL papain (Worthington) and 250 U/mL DNase I (Sigma) and then triturated to obtain a single‐cell suspension. GNPs (GFP+) and NEPs (CFP+ CD133‐ ACSA‐2‐) were then purified using FACS (ArialII, BD Bioscience). For isolation of GNPs from WT mice, the cell suspension was obtained as described above and then centrifuged through a 35%–65% Percoll gradient (Sigma). Cells from the 35%–65% interface were resuspended and cultured in NB‐B27 (Neurobasal with B27 supplement, 1 mM sodium pyruvate, 2 mM L‐glutamine, and 1% Pen/Strep). Cells were plated at 2 × 10^5^ cells/coverslip or 2 × 10^6^ cells per well in 24‐well plates precoated with PDL. NEPs followed the same culture conditions. In certain experiments, NEPs were treated with 1 μg/mL recombinant Shh‐N protein (Novoprotein) for 24 h. For primary T‐cell and microglial isolation, irradiated and nonirradiated cerebella were digested into a single‐cell suspension, and T cells (CD3+) and microglia (CD45^low^ CD11b+ F4/80+) were sorted by FACS. The fluorescence‐conjugated antibodies used in FACS sorting included anti‐CD133 (1:50, Millipore), anti‐ACSA‐2 (1:20, Miltenyi), anti‐CD3 (1:100, Biolegend), anti‐CD45 (1:100, BD Pharmingen), anti‐CD11b (1:100, Biolegend), anti‐F4/80 (1:100, Biolegend), anti‐FcR (1:50, Biolegend), and isotype rat IgG (1:100, Biolegend).

### 2.4 Immunofluorescence/immunohistochemistry and Western blotting

For immunofluorescence, cells were plated on PDL‐coated coverslips with the treatments mentioned in the text and fixed with 4% paraformaldehyde (PFA). After permeabilization with 0.1% Triton X‐100 in PBS (PBST) and blocking with 1% BSA in PBST, coverslips were incubated with primary antibodies overnight at 4°C and then incubated with fluorescence‐conjugated secondary antibodies for 2 h at room temperature. DAPI was stained at the last step for 10 min at room temperature. Four washes with PBST were performed following each staining step. Then, coverslips were mounted with Fluoromount G before being visualized using a Nikon Eclipse Ti microscope. For immunohistochemistry, cerebella were harvested and fixed overnight in 4% PFA, cryoprotected gradiently in 15% and 30% sucrose, frozen in Tissue Tek‐OCT (Sakura Finetek) and cut into 10–12 μm sagittal sections. Immunofluorescence staining for sections shared the same protocol for cytostaining described above. Hematoxylin and eosin‐stained paraffin sections were performed by Wuhan Servicebio Technology.

Western blotting was performed as regular, cerebellum tissues were lysed in RIPA buffer (Beyotime) supplemented with protease and phosphatase inhibitors (Beyotime). Total lysates containing equal amounts of protein were separated by SDS–PAGE and subsequently transferred onto PVDF membranes. Membranes were then probed with antibodies. Western blotting signals were detected by using SuperSignal West Pico Chemiluminescent substrate (Thermo).

Primary antibodies used in immunofluorescence/immunohistochemistry and Western blotting included anti‐cleaved Caspase‐3 (1:500, CST), anti‐Tuj1(1:500, Novus), anti‐phosphorylated γ‐H2AX (1:2000, Sigma), anti‐Ki67 (1:500, Abcam), anti‐NeuN (1:200, Millipore), anti‐GFP (1:500, CST), anti‐Calbindin (1:1000 CST), anti‐Lhx1 (1:500, Abcam), anti‐Shh (1:500, CST), anti‐IFN‐γR1(CD119, 1:100, BD Pharmingen), anti‐phosphorylated STAT1 (1:1000, CST), anti‐total STAT1 (1:1000, CST), anti‐Iba‐1 (1:300, Invitrogen), anti‐Nestin (1:200; R&D Systems), and anti‐GAPDH (1:2000, Sigma). The fluorescence and HRP conjugated secondary antibodies included Alexa Fluor‐594 anti‐rabbit IgG (1:200), Alexa Fluor‐594 anti‐mouse IgG (1:200), FITC Fluor‐488 anti‐rabbit IgG (1:200), FITC Fluor‐488 anti‐mouse IgG (1:200), and HRP‐anti‐rabbit IgG (1:1000) from Proteintech, Fluor 488 anti‐chicken IgY (1:200) from Invitrogen.

### 2.5 Comet assay

GNPs and NEPs were irradiated at 2 Gy and underwent a 30‐min recovery. Then cells were harvested and subjected to an alkaline comet assay by using the Comet Assay Kit (Trevigen) according to the manufacturer's protocol. After single‐cell electrophoresis on comet slides, slides were stained with ethidium bromide, and comet tail length was quantified and normalized to the nonirradiated control. A minimum of 100 cells per sample were counted.

### 2.6 Tamoxifen and vismodegib treatment

Tamoxifen (Sigma) was dissolved in corn oil (Sigma). *Nestin‐CreER* ^*T2*^ */ R26R‐GFP* mice at P4 with or without irradiation were administered tamoxifen daily by oral gavage at 0.6 mg/50 μL until the mice reached the age of P21. Vismodegib (Selleck) was dissolved in 0.9% NaCl containing 1% DMSO, 5% Tween 80, and 44% PEG 300. For vismodegib treatment, *Nestin‐CFP* mice were irradiated at P4, and 24 h after irradiation, mice were treated with daily oral gavage of 50 mg/kg vismodegib for 3 days.

### 2.7 Microarray analysis

RNAs isolated from GNPs and NEPs of naïve mice (Figure 2H), as well as NEPs of irradiated and nonirradiated mice (Figure 4A), were hybridized to Affymetrix Mouse Genome 430 2.0 arrays. Microarray data were preprocessed using robust multichip analysis. Gene ontology analysis was carried out to examine the biological functions of the differentially expressed genes using NexusExp3 software. KEGG functional enrichment analysis was conducted based on the R package Cluster Profiler, and the results of enrichment analysis were visualized via the R package enrich plot. A *p* value <0.05 was set as the criterion.

### 2.8 EdU incorporation and detection

Mice cerebella were irradiated at P4 and then collected for frozen section preparation at different hours post irradiation. Six hours before cerebellum collection, EdU (Beyotime) was administered by intraperitoneal injection to mice at 100 mg/kg, and then the sections were stained for EdU.

### 2.9 ELISA (enzyme‐linked immunosorbent assay)

Irradiated and nonirradiated cerebella were homogenized and centrifuged, and the supernatant was collected to perform ELISA. The total protein concentrations of the samples were detected with a BCA Protein Assay Kit (Beyotime) and adjusted to be equal in each group. To detect IFN‐γ, a 96‐well plate was precoated with purified anti‐mouse IFN‐γ monoclonal antibody (1:500, clone R4‐6A2, BD Pharmingen) overnight at 4°C. The plate was washed with PBST (PBS containing 0.05% Tween‐20) and blocked with 1% BSA in PBST. Samples and standards (recombinant mouse IFN‐γ protein, BD Pharmingen) were added to the wells and incubated for 1 h at room temperature. Biotinylated anti‐mouse IFN‐γ antibody (1:500, clone XMG1.2, BD Pharmingen) was later used to bind to IFN‐γ antigen for 1 h at room temperature. Then, streptavidin‐HRP (1:1000, BD Pharmingen) was added and incubated for 30 min. Finally, the substrate TMB (Beyotime) was added for color development, and the reaction was stopped by 1 M H_2_SO_4_ for 10 min. The optical density (OD) in each well was read with a microplate reader at 450 nm. The IFN‐γ level was calculated according to the standard curve.

### 2.10 Real‐time quantitative PCR (qPCR)

Total RNA was extracted from cell lysate or tissue homogenate using TRIzol reagent (Sigma) in RNase‐free conditions, and the purity of RNA was detected with a Nanodrop 2000 spectrophotometer (Thermo). cDNA was synthesized using oligo (dT) and Superscript II reverse transcriptase (Invitrogen). qPCR was performed in triplicate using SYBR qPCR Master Mix (Vazyme) and the ABI 7500 TaqMan Real‐Time PCR Detection System. The differences in mRNA expression were calculated by the 2^−ΔΔCt^ method. The primers (all for mouse species) used in the experiments included *GAPDH* (forward: 5‐CATCACTGCCACCCAGAAGACTG‐3; reverse: 5‐ATGCCAGTGAGCTTCCCGTTCAG‐3); *Shh* (forward: 5‐GGATGAGGAAAACACGGGAGCA‐3; reverse: 5‐TCATCCCAGCCCTCGGTCACT‐3); *IFN‐γ* (forward: 5‐TGAGCTCATTGAATGCTTGG‐3; reverse: 5‐ACAGCAAGGCGAAAAAGGAT‐3); *IL‐1α* (forward: 5‐CCAGAAGAAAATGAGGTCGG‐3); reverse: 5‐CCAGAAGAAAATGAGGTCGG‐3); *IL‐6* (forward: 5‐CTCTGCAAGAGACTTCCATCCAGT‐3; reverse: 5‐GAAGTAGGGAAGGCCGTGG‐3); *Gli1* (forward: 5‐CTCAAACTGCCCAGCTTAACCC‐3; reverse: 5‐TGCGGCTGACTGTGTAAGCAGA‐3); *Ptc2* (forward: 5‐CTCCGCACCTCATATCCTAGC‐3; reverse: 5‐TCCCAGGAAGAGCACTTTGC‐3); and *Sfrp1* (forward: 5‐CAATACCACGGAAGCCTCTAAGC‐3; reverse: 5‐GCTTGCACAGAGATGTTCAATG‐3).

### 2.11 Slice culture

Cerebellum slice cultures were performed according to a previous publication.^23^ Irradiated or nonirradiated cerebella were embedded in 3% tissue culture grade agarose, and 300 μm sagittal slices were cut using a VT100S vibrating microtome (Leica). Slices were then transferred to a 0.4 μm Nuclepore membrane (Millicell) at the interface between air and culture medium (NB‐B27 medium) in a 6‐well culture plate and incubated at 37°C in 5% CO_2_. In certain experiments, cerebellum slices derived from nonirradiated mice were treated with 200 U/mL recombinant mouse IFN‐γ (Peprotech) for 24 h during culture, and then tissue lysates were prepared for Western blotting and qPCR. Cerebellum slices derived from irradiated mice 12 h post irradiation were treated with an IFN‐γ neutralizing antibody (clone R4‐6A2, BioXcell) or isotype control rat IgG1 for 24 h at 10 μg/mL, and then tissue lysates and frozen sections were prepared for Western blotting and immunohistochemistry, respectively.

### 2.12 Statistical analysis

Experimental data were analyzed using GraphPad Prism software. The Shapiro–Wilk test was used to test for data distribution normality. Unpaired *Student's t* test was used to calculate the difference when data exhibit Gaussian distribution. Data that do not exhibit Gaussian distribution was analyzed via a non‐parametric *Student's t* test. Differences were considered to be significant when their value was less than 0.05 (*p* < 0.05). Data are expressed as the mean ± SEM.

## 3 RESULTS

### 3.1 NEPs were more resistant to irradiation than GNPs

**FIGURE 1:**
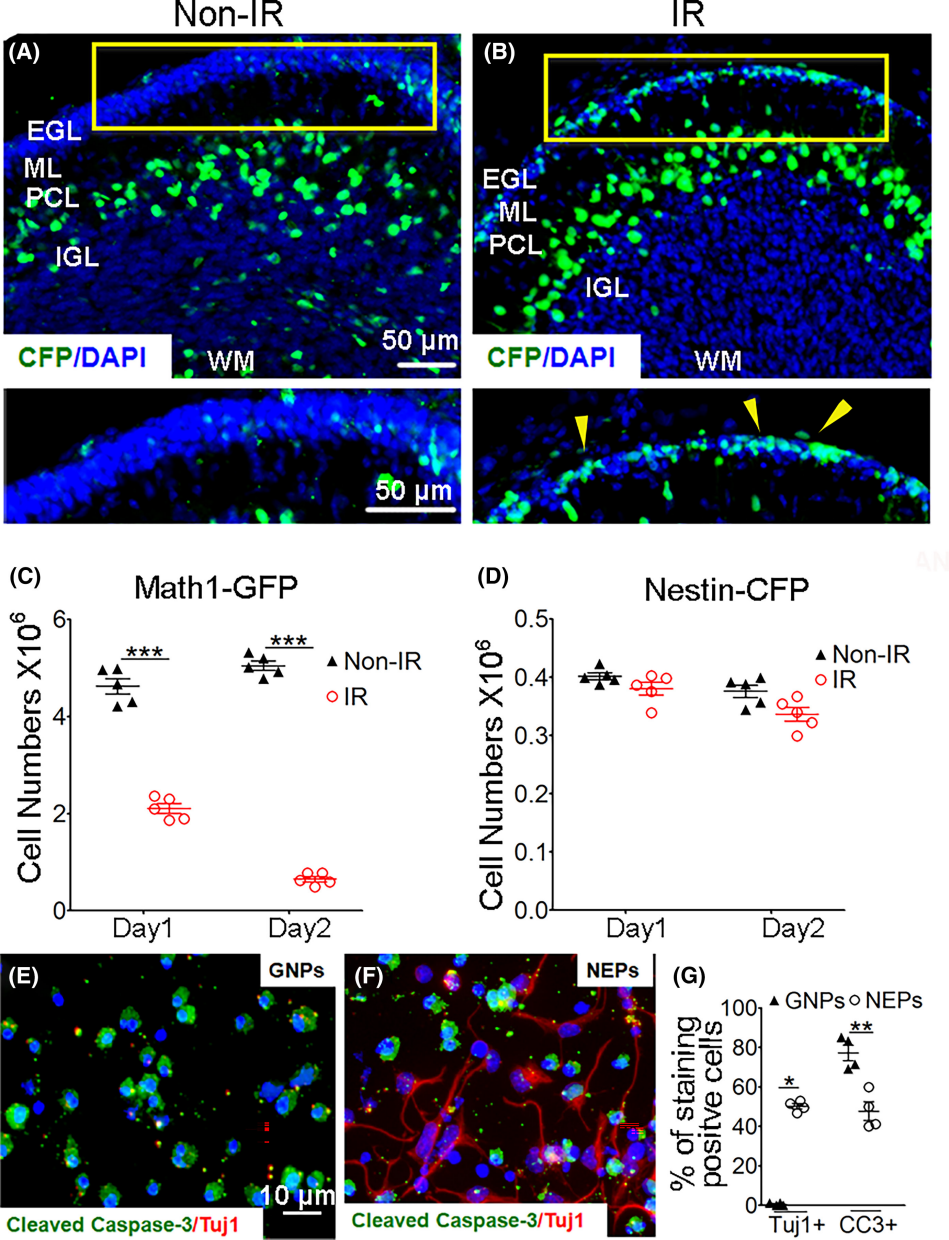
Nestin‐expressing progenitors (NEPs) were more resistant to irradiation than granule neuron precursors (GNPs). (A, B) The cerebella of *Nestin‐CFP* mice were irradiated with a 4 Gy dose (IR) or not (Non‐IR) at P4, and the cerebellum sections were collected 2 days later to perform immunostaining for GFP or CFP. DAPI counterstained the nuclei. (C, D) *Math1‐GFP* (C) and *Nestin‐CFP* (D) mice were irradiated on the cerebellum with a 4 Gy dose at P4, 1 or 2 days post irradiation, the numbers of GNPs (GFP+) and NEPs (CFP+ CD133‐ACSA‐2‐) were quantified by FACS. *n* = 5 mice in panel (A–D). (E, F) GNPs and NEPs were sorted from the EGL of nonirradiated *Math1‐GFP* and *Nestin‐CFP* mice at P4, respectively, and seeded in culture plates. Then, the cells were irradiated with a single dose of 2 Gy. Twenty‐four hours after irradiation, immunostaining was performed to detect the cell apoptosis marker cleaved caspase‐3 (CC3) and differentiation marker Tuj1. (G) Statistical quantification of the percentage of CC3+ and Tuj1+ cells in panels (E, F). **p < 0.01; ***p < 0.001.

Our previous studies showed that NEPs are quiescent in normal cerebellum development but are more tumorigenic in Shh‐medulloblastoma than GNPs in *Ptc1* gene deficient mice.^21^ These findings lead us to speculate that NEPs may react to radiotherapy‐induced damage differently than GNPs. To address this question, *Nestin‐CFP* transgenic mice, in which the mouse *Nestin* promoter directs the expression of a cyan fluorescent protein (CFP) with a nuclear localization signal, and *Math1‐GFP* mice were used to establish a radiotherapy‐induced cerebellum injury model. In the cerebellum of *Nestin‐CFP* mice, NEPs reside in the deep part of the EGL (Figure 1A), and there are also Nestin‐CFP‐positive cells located in the ML and WM, which are astrocytes and neural stem cells, respectively.^21^ Mouse cerebella were irradiated with a single dose of 4 Gy on P4, and at P6, the irradiated cerebella were harvested for immunohistochemistry analysis. Cerebella from *Nestin‐CFP* mice at P6 without irradiation were used as controls. The results showed that irradiation eliminated the majority of GNPs in the cerebellum, resulting in a much thinner EGL in the irradiated cerebellum than in the naïve cerebellum (Figure 1A,B). This finding is consistent with previous reports that cerebellar GNPs are very sensitive to irradiation. However, cells that survived irradiation were enriched by CFP+ cells, suggesting that NEPs may be more radioresistant than conventional GNPs. To further confirm this, we also irradiated the cerebella of *Math1‐GFP* mice in which conventional GNPs (specifically expressing Math1) are positive for GFP at P4. We then isolated cells from the cerebella at 1 and 2 days following irradiation and analyzed the number of GNPs (GFP+) and NEPs (CFP+ CD133‐ ACSA‐2‐) per cerebellum by FACS. CD133 and ACSA‐2 are the cell surface markers for neural stem cells and astrocytes, respectively, and they were used here to exclude these two Nestin/CFP+ populations in *Nestin‐CFP* mice. Compared with that in the nonirradiated cerebella, the number of GNPs was markedly reduced in the cerebella following irradiation. As a comparison, the number of NEPs remained indistinguishable between the irradiated cerebellar and nonirradiated ones, indicating that NEPs are more resistant to irradiation than GNPs (Figure 1C,D). In addition, we purified GNPs and NEPs from the cerebella of nonirradiated *Math1‐GFP* and *Nestin‐CFP* mice, respectively, and treated both cell populations with 2 Gy irradiation in vitro. Forty‐eight hours after irradiation, cells were harvested to detect apoptosis and differentiation by immunocytochemistry. As shown in Figure 1E–G, most GNPs were positive for cleaved caspase‐3 and exhibited condensed nuclei, suggesting that they were undergoing apoptosis. However, only a proportion of NEPs were found to be apoptotic; instead, the majority of NEPs started their differentiation in vitro, as reflected by the intensive expression of Tuj1, a maker of granule neurons. The above data demonstrate that NEPs represent a radioresistant neuronal population in the developing cerebellum.

### 3.2 NEPs resembled GNPs in DNA damage following irradiation but downregulated DNA damage response and repair (DDR) genes

**FIGURE 2:**
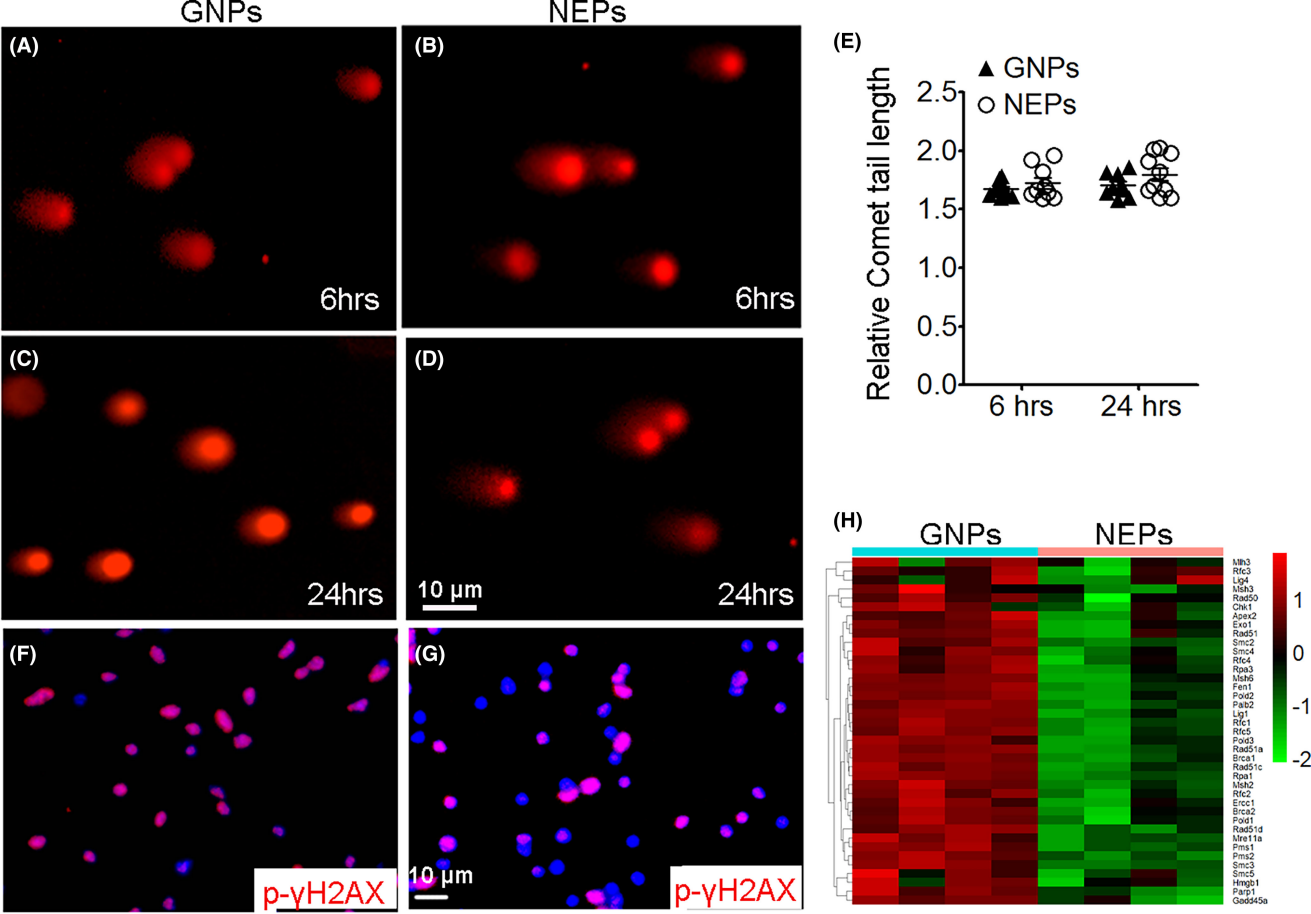
Nestin‐expressing progenitors (NEPs) received similar DNA damage as granule neuron precursors (GNPs) to irradiation but had a downregulated damage response and repair (DDR). GNPs and NEPs were isolated from the cerebella of nonirradiated *Math1‐GFP* and *Nestin‐CFP* mice, respectively, and were irradiated in vitro with a 2 Gy dose (A–G) or not (H). (A–D) Comet assay was performed at 6 or 24 h after irradiation. (E) Statistical quantification of the relative comet tail length in panels (A–D). (F, G) Phosphorylated γH2AX immunostaining was carried out 24 h post irradiation, and DAPI was used to counterstain the nuclei. (H) mRNA microarray was performed and analyzed to compare the DDR gene expression profiles in nonirradiated NEPs and GNPs. The heatmap shows the downregulation of DDR genes in NEPs compared with GNPs.

To determine the reason for the radioresistance of NEPs, we examined the extent of DNA damage in GNPs and NEPs after irradiation by comet tailing assay. Purified GNPs and NEPs from nonirradiated mice were plated in vitro and treated with 2 Gy irradiation. Irradiated GNPs and NEPs were harvested for the comet tailing assay. As shown in Figure 2A–E, comparable lengths of DNA tails were observed in GNPs and NEPs at 6 and 24 h following irradiation, suggesting that irradiation caused similar extents of DNA damage in GNPs and NEPs. We next examined the difference in the DDR of NEPs and GNPs after irradiation. For this purpose, we harvested NEPs and GNPs 24 h after 2 Gy irradiation and examined the phosphorylation of γ‐H2AX, a marker of DDR. The results showed that the phosphorylation of γ‐H2AX‐positive cells in irradiated NEPs was less than that in GNPs (Figure 2F,G). Moreover, mRNA microarray was performed to determine the DDR gene expression profile in nonirradiated NEPs and GNPs. We observed markedly downregulated expression of DDR genes in NEPs compared with GNPs (Figure 2H), suggesting the decreased capacity of DNA repair of NEPs, which probably allow them to tolerate DNA damage by irradiation. All these data suggest that in response to irradiation, a downregulated DDR probably render NEPs resistant to irradiation.

### 3.3 NEPs regenerated to repopulate cerebellar neuronal populations after irradiation

**FIGURE 3:**
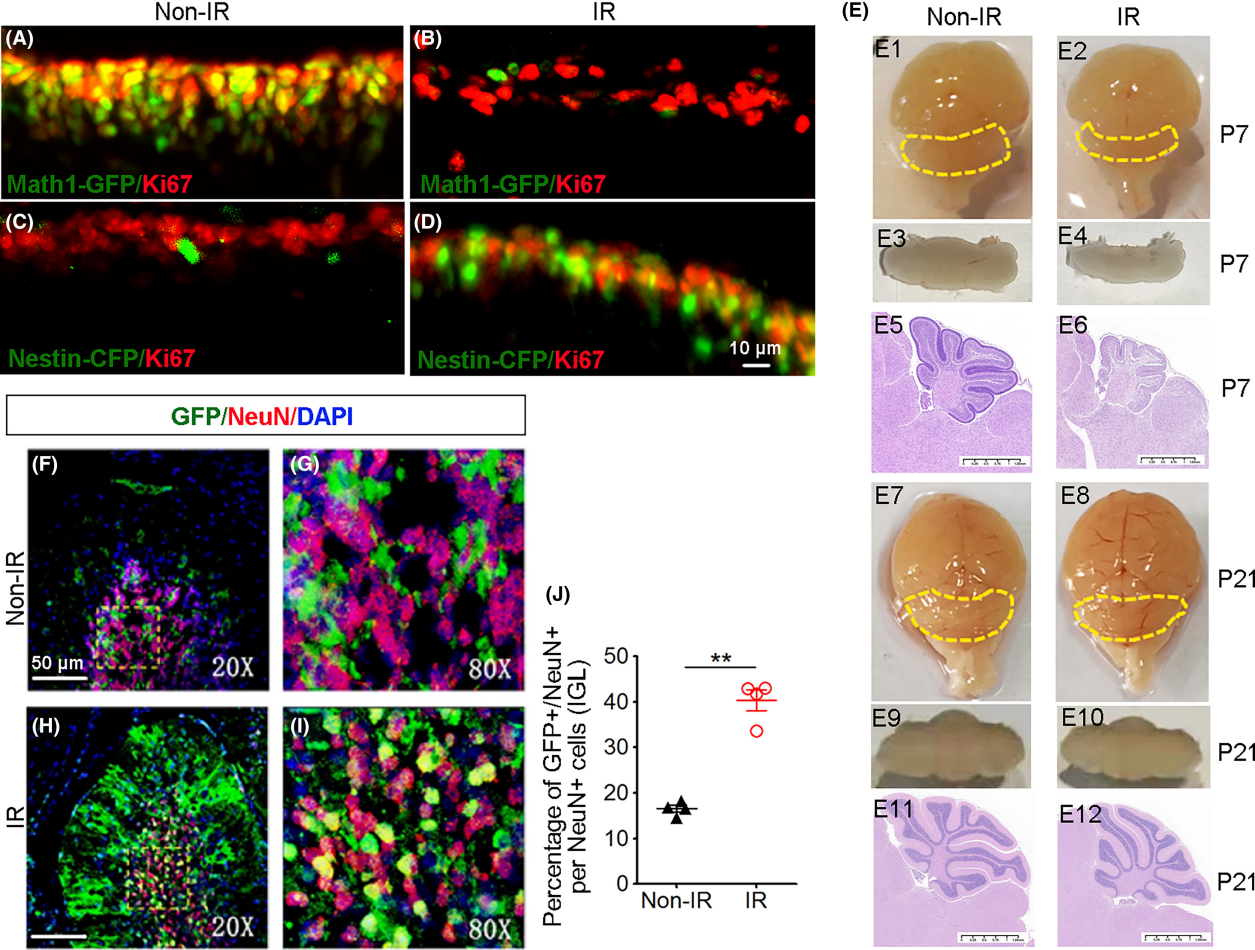
Nestin‐expressing progenitors (NEPs) regenerated to repopulate cerebellar neuronal populations following irradiation. (A–D) *Math1‐GFP* (A, B) and *Nestin‐CFP* mice (C, D) were irradiated (B, D) with 4 Gy at P4 or not (A, C), and the cerebellum sections were collected at P8 followed by immunostaining for GFP/CFP and the proliferation marker Ki67. (E) Wild‐type (WT) mice were irradiated or not at P4, and the brains or cerebella were collected for picture (upper and middle panels) and hematoxylin and eosin stained mid sagittal sections (lower panel) at P7 and P21. (F–I) *Nestin‐CreER* ^*T2*^ */R26R‐GFP* mice were irradiated (H, I) or not (F, G) at P4 and were treated with tamoxifen daily until the mice reached P21. Then, the cerebellum was dissected and sectioned for immunostaining for GFP and NeuN, DAPI counterstained the nuclei (G, I: zoom in of F, H). (J) Quantification of GFP/NeuN double‐positive cell percentages among total NeuN‐positive cells in panels (F–I). *n* = 4 mice. **p < 0.01.

Next, to investigate whether radioresistant NEPs have regeneration capacity after irradiation, we evaluated their proliferation in the cerebellar EGL by immunohistochemistry. Briefly, cerebella of *Math1‐GFP* mice or *Nestin‐CFP* mice were irradiated at P4 and collected at P8 for immunohistochemistry. Cerebella from *Math1‐GFP* mice or *Nestin‐CFP* mice at P8 without irradiation were also harvested as controls. As expected, conventional GNPs (GFP+) in the EGL were highly proliferative (Ki67+) in nonirradiated *Math1‐GFP* cerebella (Figure 3A). The majority of GNPs were ablated by irradiation, resulting in almost no GFP‐positive cells remaining in the EGL following irradiation, and they did not proliferate after irradiation (Figure 3B). Consistent with our previous report, NEPs were predominantly quiescent in naïve *Nestin‐CFP* cerebellum at P8, which resided in the deep part of the EGL (Figure 3C). However, the majority of NEPs were positive for Ki67 4 days post irradiation (Figure 3D), suggesting that NEPs initiated their proliferation in response to irradiation. Then, we checked the size of the cerebella with/without irradiation at P7 and P21 by cerebellum appearance and hematoxylin and eosin‐stained mid sagittal sections. We found that the irradiated cerebella were much smaller than the nonirradiated ones 3 days after irradiation, while the size of the irradiated cerebella almost recovered to that of the nonirradiated cerebella at P21 (Figure 3E). The results suggest that NEPs proliferated and the regeneration of the cerebellum occurred after irradiation. To determine whether those proliferative NEPs contribute to neurogenesis in the irradiated cerebellum, we crossed *Nestin‐CreER* ^*T2*^ mice expressing inducible Cre recombinase in Nestin+ cells with *R26R‐GFP* mice in which cells permanently express GFP after Cre recombination. *Nestin‐CreER* ^*T2*^ */R26R‐GFP* mice were orally treated with tamoxifen with or without irradiation at P4. The cerebella were harvested at P21 to analyze the fate of NEPs following irradiation. As shown in Figure 3F–J, in the IGL of the cerebellum, approximately 40% of mature granule neurons (NeuN+) were positive for GFP. As a comparison, in the *Nestin‐CreER* ^*T2*^ */R26R‐GFP* cerebellum without irradiation, GFP+ cells accounted for less than 20% of mature granule neurons, suggesting that normally a small proportion of granule neurons originate from NEPs, whereas in the situation of irradiation‐induced cerebellar injury, NEPs proliferate and differentiate into mature granule neurons. These data showed increased granule neurons derived from NEPs after irradiation, indicating that NEPs participate in the regeneration of the irradiated cerebellum.

### 3.4 Regeneration of NEPs relied on the Shh signaling pathway

**FIGURE 4:**
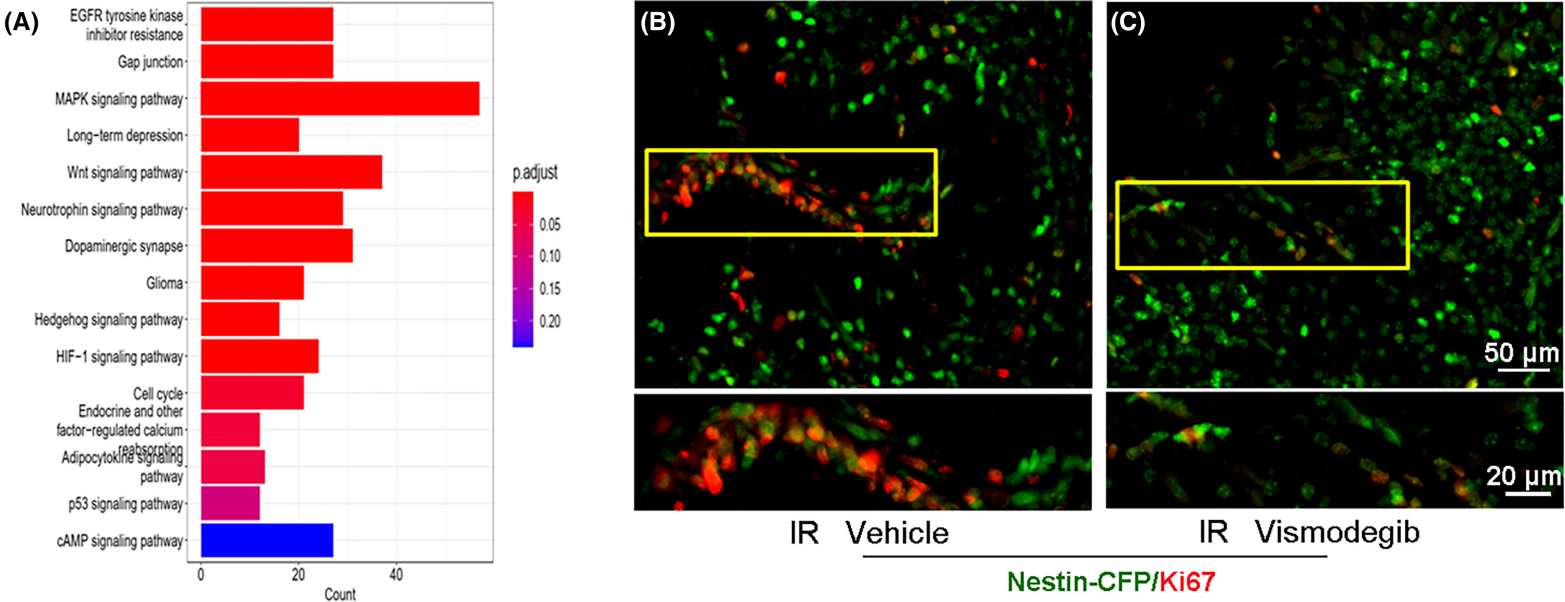
Regeneration of Nestin‐expressing progenitors (NEPs) relied on the Shh signaling pathway. (A) Cerebella of *Nestin‐CFP* mice were irradiated with a 4 Gy dose at P4 and were collected at P6 for NEP isolation. NEPs derived from naïve P6 mice served as controls. Then, mRNA was extracted from NEPs to perform microarray. KEGG functional enrichment analysis showed the top 15 activated signaling pathways in NEPs from irradiated cerebellum compared with nonirradiated controls. (B, C) *Nestin‐CFP* mice were irradiated with 4 Gy at P4, and vismodegib (50 mg/kg) or vehicle was administered to the irradiated mice by oral gavage 1 day later for three consecutive days. Then, cerebellum sections were collected and immunostained for GFP and Ki67. *n* = 4 mice in panel (B, C).

To further investigate the molecular basis for NEP regeneration after irradiation, we harvested NEPs from *Nestin‐CFP* cerebella 2 days after irradiation at P4, since at this time point, NEPs started to proliferate in response to irradiation. Naïve NEPs from P6 *Nestin‐CFP* mice without irradiation were collected as controls. Then, mRNA was extracted from proliferative NEPs and naïve NEPs, which were used for microarray analysis. Then, KEGG functional enrichment analysis was performed. The top 15 activated signaling pathways were enriched (Figure 4A). Several signaling pathways involved in cell proliferation were activated in NEPs derived from irradiated mice compared with their naïve counterparts, such as the MAPK and WNT pathways. Among them, the Shh signaling pathway was also activated. To confirm whether Shh signaling is involved in NEP regeneration, we next tested whether such regeneration can be repressed by inhibition of the Shh pathway. For this purpose, 24 h after irradiation, *Nestin‐CFP* mice were treated with daily oral gavage of vismodegib, an established antagonist of the Shh signaling pathway component, Smoothened. Then, the cerebella were harvested at P8 to examine NEP proliferation by immunohistochemistry. As shown in Figure 4B,C, the proliferation of NEPs was markedly repressed by vismodegib treatment. These data indicate that Shh signaling is required for NEP regeneration after irradiation.

### 3.5 Augmented Shh ligand was provided by Purkinje cells following irradiation

**FIGURE 5:**
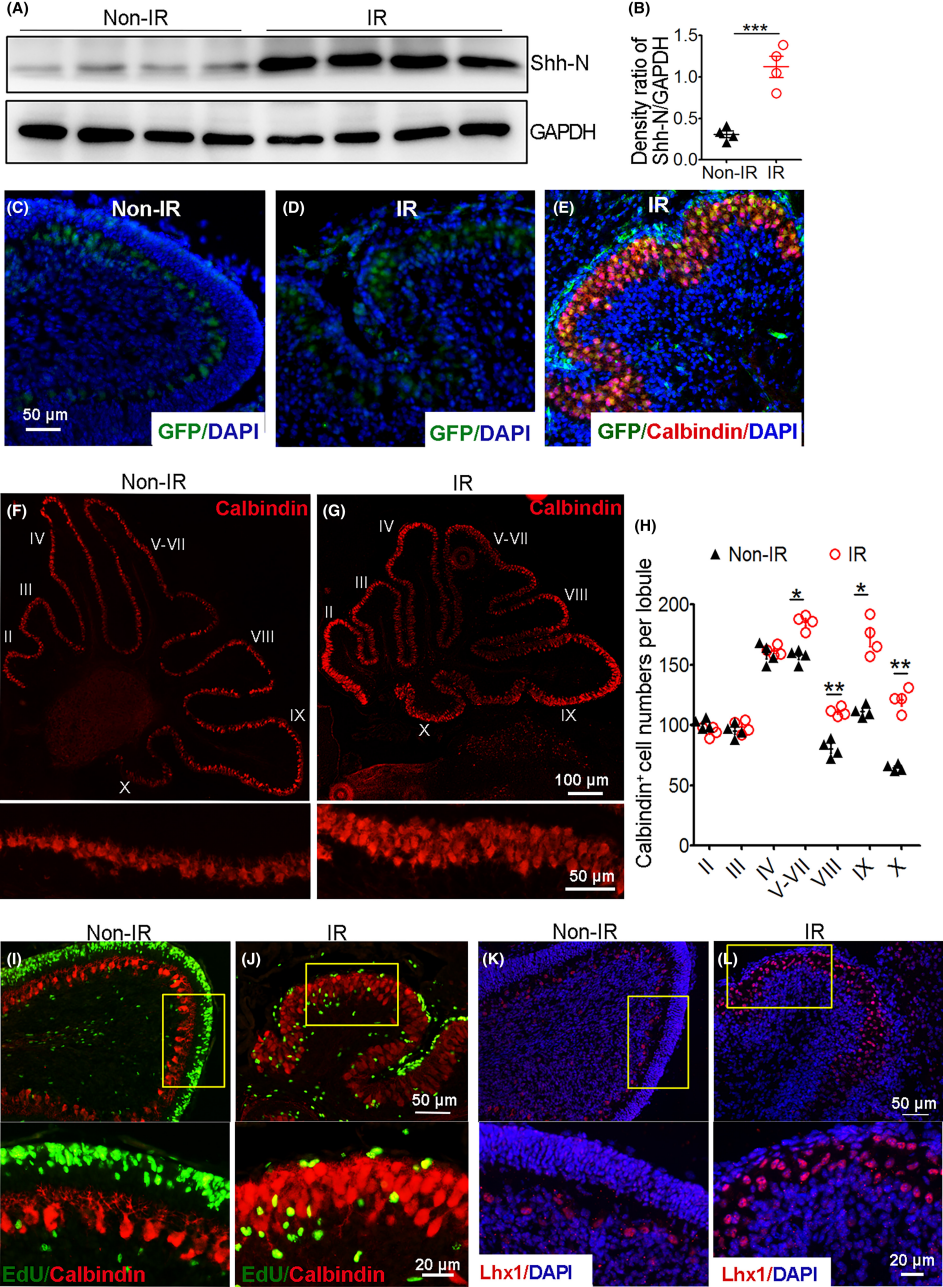
Augmented Shh ligand was provided by Purkinje cells following irradiation. Wild‐type (WT) (A, B, F–L) and *Shh‐Cre‐GFP* (C–E) mice were irradiated on the cerebellum with 4 Gy or not at P4. (A) Two days post irradiation, cerebella were harvested, and the tissue lysates were prepared for Shh‐N protein evaluation by Western blotting. GAPDH served as the protein sample loading control. (B) Statistical quantification of the density ratio of Shh‐N/GAPDH in panel (A). (C–E) *Shh‐Cre‐GFP* mice were irradiated (D, E) or not (C), and cerebellum sections were obtained 2 days later and immunostained for GFP or together with the Purkinje cell marker, Calbindin. DAPI was used to stain the nuclei. (F, G) Mid sagittal cerebellum sections from irradiated (F) and nonirradiated (G) mice were immunostained with Calbindin. (H) Quantification of Calbindin‐positive cell numbers in each lobule of the irradiated and nonirradiated cerebella in panel (F, G). (I, J) The cerebella of mice with or without irradiation were collected 48 h post irradiation, EdU was administered to mice 6 h before harvesting the cerebella, and the cerebellum sections were immunostained for Calbindin and EdU. (K, L) Twenty‐four hours after irradiation, cerebellum sections were obtained and immunostained for the Purkinje cell precursor marker Lhx1, DAPI counterstained the nuclei. *n* = 4 mice. *p < 0.05; **p < 0.01.

Since the Shh signaling pathway is involved in NEP regeneration after irradiation, we wondered which step of the pathway was altered to contribute to NEP regeneration. To address this question, we first tested the trigger of the Shh signaling pathway, Shh ligand, in the cerebella of irradiated mice 2 days post irradiation. The Western blotting results showed that the protein level of active Shh ligand (N‐terminal of full‐length Shh, Shh‐N) was augmented in the irradiated cerebella compared with that in the naïve cerebella (Figure 5A,B). The mRNA level of Shh was also evaluated and shared a similar pattern (Figure S1). These results indicate that Shh ligand expression was upregulated in the cerebellum post irradiation. To confirm whether NEPs can respond to the Shh ligand to proliferate, NEPs were isolated from irradiated *Nestin‐CFP* mice at 2 days post irradiation and cultured in vitro for 24 h with recombinant Shh‐N protein or vehicle control. The results showed that NEPs proliferated with Shh‐N incubation but not vehicle control (Figure S2), suggesting that NEP proliferation in vivo via Shh signaling after irradiation probably due to their responding to the enhanced Shh ligand. Then, considering that Purkinje cells and astrocytes are the main source of Shh ligand in normal cerebellum development and Shh‐medulloblastoma tumorigenesis, respectively,^13^, ^24^ we then identified the cell source of Shh ligand after irradiation in our model. For this purpose, *Shh‐Cre‐GFP* transgenic mice, in which cells expressing Shh are GFP positive, were irradiated on cerebella at P4, and the cerebella were collected at P6 for immunohistochemistry analysis. Cerebella from nonirradiated P6 *Shh‐Cre‐GFP* mice were also collected as controls. We observed that Shh‐producing cells (GFP+ cells) were significantly increased in the irradiated cerebella compared with the nonirradiated ones, and the Shh‐producing cells resided in the Purkinje cell layer (Figure 5C,D). Then, the cerebellar sections were counterstained with the Purkinje cell marker, Calbindin (Figure 5E) and astrocyte marker GFAP (data not shown), and it was confirmed that Purkinje cells were the main source of the Shh ligand after irradiation. Meanwhile, as shown in Figure 5E, The number of Purkinje cells was increased following cerebellar irradiation. To confirm this, Purkinje cell numbers in the mid sagittal cerebellum sections were counted according to Calbindin‐positive staining. As shown in Figure 5F–H, Purkinje cell numbers in cerebellar lobules II–IV were comparable between the irradiated and nonirradiated cerebella, while their numbers in lobules V–X were significantly higher in the irradiated cerebella than in the naïve controls, although the size of the irradiated cerebella was smaller. Then, we wondered if Purkinje cells proliferated after irradiation. To confirm this, WT mice were irradiated and then collected for section preparation at 12, 24, 48, and 72 h post irradiation. EdU was administered to mice 6 h before cerebellum collection, and then the sections were stained for EdU and Calbindin. A parallel experiment was performed with naïve mice as a control. As shown in Figure 5I–L and Figure S3, the number of Purkinje cells increased significantly beginning 24 h post irradiation, but they did not show signs of proliferation, as evaluated by EdU incorporation at these time points. These results led us to speculate that the increase in Purkinje cells might be due to differentiation from their precursors. We then immunostained the sections above for the Purkinje cell precursor marker, Lhx1 and found that the number of Purkinje cell precursors in the irradiated cerebellum increased at 12 h and decreased beginning 24 h after irradiation (Figure S3 and Figure 5K,L). All the data above indicate that Shh ligand was upregulated in the Purkinje cells of the irradiated cerebellum. And the number of Purkinje cells was increased in lobule V–X of the irradiated cerebellum, which might be differentiated from their precursors.

### 3.6 IFN‐γ stimulated Shh ligand expression following irradiation, contributing to NEP proliferation

**FIGURE 6:**
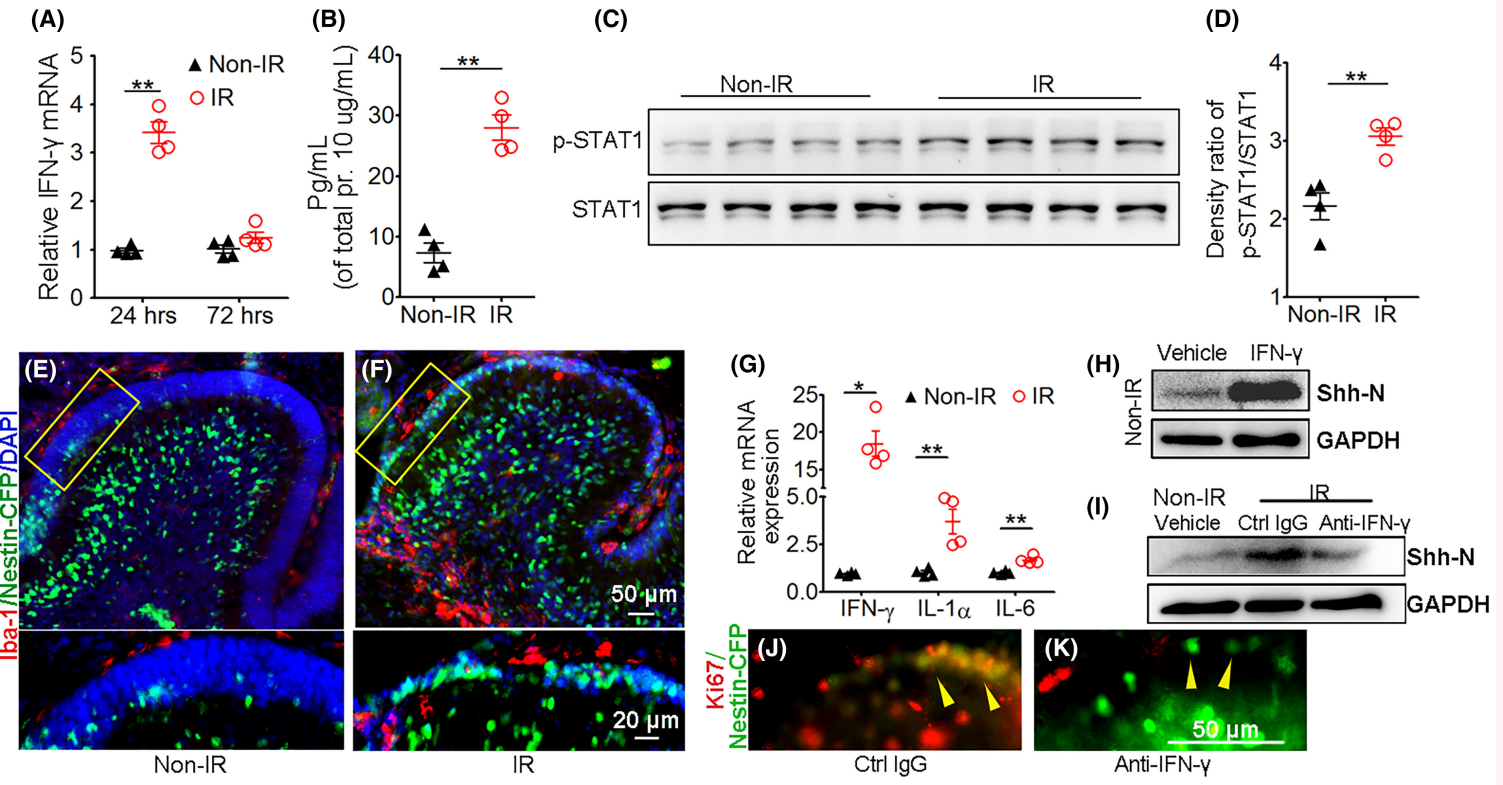
Increased IFN‐γ stimulated Shh ligand expression following irradiation and contributed to Nestin‐expressing progenitor (NEP) proliferation. Wild‐type (WT) (A–D, G–I) and *Nestin‐CFP* (E, F, J, K) mice were irradiated with 4 Gy or not at P4. (A) Twenty‐four or 72 h after irradiation, cerebellum tissue was harvested for RNA isolation. The mRNA levels of IFN‐γ were determined by qPCR. (B, C) Twenty‐four hours after irradiation, cerebellum tissue was collected and homogenized, the protein levels of IFN‐γ in the cerebellum were evaluated by ELISA (B), and the phosphorylated STAT1 (p‐STAT1) and total STAT1 protein levels were assessed by Western blotting (C). (D) Statistical quantification of the density ratio of phosphorylated STAT1/ total STAT1 in panel (C). (E, F) Twenty‐four hours after irradiation, irradiated (F) and control (E) cerebellum sections were prepared and immunostained for CFP and the microglial marker Iba‐1, and DAPI counterstained the nuclei. (G) Microglia were sorted by FACS from irradiated or control mice 24 h after irradiation for RNA extraction, and the mRNA levels of the indicated cytokines were determined by qPCR. *p < 0.05; **p < 0.01. (H) Cerebella of naïve WT mice at P4 were collected and sliced in agarose gel and then subjected to slice culture on a semipermeable membrane with or without 200 U/mL recombinant IFN‐γ for 24 h. Then, the tissue was collected for Shh protein evaluation by Western blotting. (I) WT mice were irradiated at P4, 12 h after irradiation, cerebella were harvested for slice culture, and during slice culture, 10 μg/mL neutralizing anti‐IFN‐γ antibody or control rat IgG was administered for 24 h, slices from nonirradiated cerebellum treated with vehicle served as a control. Then, the slice tissue was collected to evaluate the Shh protein levels by Western blotting, GAPDH served as the sample loading control. (J, K) Twelve hours after *Nestin‐CFP* mice were irradiated; slice culture was performed and cultured for 48 h. During the culture, 10 μg/mL neutralizing anti‐IFN‐γ antibody or control rat IgG was administered to the culture. Then, the slice sections were prepared and immunostained for Ki67 and CFP. *n* = 4 mice.

We next sought to investigate the mechanisms by which the Shh ligand was upregulated in the irradiated cerebellum. It was reported by some publications that Shh ligand expression can be induced by IFN‐γ in neural cells under certain conditions.^25^, ^26^ This led us to speculate that IFN‐γ may also contribute to Shh ligand production by Purkinje cells following irradiation. First, we evaluated the mRNA and protein levels of IFN‐γ in the cerebella with or without irradiation. As shown in Figure 6A,B, the mRNA level of IFN‐γ in the irradiated cerebellum 24 h post irradiation was significantly upregulated compared with that in the nonirradiated cerebellum, while IFN‐γ expression showed no difference between the irradiated cerebellum and the control cerebellum when they were collected 72 h after irradiation, indicating that IFN‐γ expression was induced transiently by irradiation. Meanwhile, the protein level of IFN‐γ in the cerebella 24 h after irradiation was also increased compared with the control. Then, we tested whether the downstream signal of IFN‐γ was activated in the irradiated cerebellum. As shown in Figure 6C,D, the phosphorylation of STAT1 was increased in the irradiated cerebellum 24 h after irradiation, indicating that the downstream signal of IFN‐γ was activated in this circumstance. Next, we tried to identify the cell source of the increased IFN‐γ in the irradiated cerebellum. Since T cells are known as the main source of IFN‐γ, we tested the T‐cell composition in the cerebellum by FACS. As shown in Figure S4A, T cells (CD3+) constituted less than 1% of the cerebellum even with irradiation. T cells from irradiated and nonirradiated cerebella 24 h after irradiation were sorted for RNA extraction to evaluate IFN‐γ mRNA expression. And we found that IFN‐γ expression was not altered in T cells based on irradiation (Figure S4B). Considering that microglia, the immune cells in the brain, are reported to produce IFN‐γ in inflammatory status^27^, ^28^, ^29^ and become activated in response to irradiation,^30^, ^31^ we then checked the presence of microglia in the irradiated cerebellum. Cerebella of *Nestin‐CFP* mice were irradiated at P4 and harvested for frozen section preparation 24 h after irradiation, and the sections were immunostained with the microglia marker, Iba‐1. The results showed that more microglia accumulated around the EGL region after irradiation (Figure 6E,F). To confirm whether the accumulated microglia in the irradiated cerebellum could produce more IFN‐γ, microglia were sorted from the irradiated and nonirradiated cerebella 24 h post irradiation, and then qPCR was performed to determine IFN‐γ expression. The results showed that IFN‐γ expression in microglia derived from the irradiated cerebellum increased significantly compared with that in nonirradiated controls (Figure 6G). Meanwhile, microglial activation markers such as IL‐1α and IL‐6 were also upregulated in microglia derived from the irradiated cerebellum. These results indicate that microglia in the cerebellum were activated by irradiation and expressed more IFN‐γ. We then further studied the contribution of IFN‐γ to Shh ligand production in the irradiated cerebellum. Since we demonstrated that the augmented Shh ligand was mainly from Purkinje cells, we first detected the presence of IFN‐γ receptors on Purkinje cells in the naïve cerebellum (Figure S5). Because it is infeasible to isolate primary Purkinje cells from the murine cerebellum, we chose to use a semi in vivo method, slice culture, which maintains the cell and molecule bioactivity during culture in plates, to perform the investigation. Naïve cerebella were collected from P4 WT mice and subjected to slice culture with exogenous IFN‐γ (200 U/mL) for 24 h. Then, the slice tissue was collected to determine the protein levels of the Shh ligand by Western blotting. As shown in Figure 6H, IFN‐γ treatment significantly increased the Shh ligand protein level compared with vehicle treatment. Meanwhile, the treatment induced the expression of the Shh signal pathway target genes including *Gli1*, *Ptc2*, and *Sfrp1*, indicating that the Shh signal pathway is activated by IFN‐γ in current system (Figure S6). We also performed local IFN‐γ injection into the cerebellum of *Shh‐Cre‐GFP* mice and found that IFN‐γ injection increased Shh/GFP+ cell numbers compared with vehicle injection (Figure S7A–C). Then, to further confirm whether IFN‐γ contributed to irradiation‐induced Shh ligand augmentation, irradiated cerebella 12 h post irradiation were also collected to perform slice culture experiments. During the 24‐h culture, an IFN‐γ neutralizing antibody was added to the culture medium at a 10 μg/mL dose, and purified rat IgG served as a control. As shown in Figure 6I, Shh levels in control IgG‐treated slice culture samples derived from irradiated cerebella were augmented significantly compared with those derived from nonirradiated cerebella, whereas treatment with IFN‐γ neutralizing antibody dampened the augmentation. These results suggest that IFN‐γ contributed to the increased Shh ligand production in the irradiated cerebellum. Furthermore, to determine whether IFN‐γ could promote NEP proliferation in the irradiated cerebellum, the cerebella of *Nestin‐CFP* mice were irradiated and collected for slice culture 12 h later, and the slice culture was treated with IFN‐γ neutralizing antibody or control IgG for 48 h. Then, the slices were immunostained for Ki67 and CFP to detect NEP proliferation. The results showed that NEP proliferated during slice culture, while IFN‐γ neutralizing antibody treatment inhibited their proliferation (Figure 6J,K). Similarly, administration of IFN‐γ by intracerebellum injection into naïve *Nestin‐CFP* mice also increased NEP proliferation in vivo (Figure S7D–F). Moreover, INF‐γ^null/null^ mice were irradiated at P4, and the cerebellum sections were prepared 4 days later to detect the EGL regeneration. Although NEPs could not be clearly identified by immunofluorescence staining for Nestin in INF‐γ^null/null^ mice since the abundant glial cells highly express Nestin with cytoplasm and cell fiber distribution, we did see that the thickness of the EGL as well as the proliferating cell number in the EGL was much lower in irradiated INF‐γ^null/null^ mice compared with WT controls (Figure S8). All the results above suggest that the increased IFN‐γ production following cerebellum irradiation enhanced Shh ligand levels and contributed to NEP proliferation.

## 4 DISCUSSION

As one of the main therapeutic methods for brain tumors, radiotherapy shows potency but always causes severe side effects, especially in the treatment of pediatric brain tumors. For example, in the most common pediatric brain tumor, cerebellar medulloblastoma, radio‐ and chemotherapy will induce cognitive disorders, endocrine dysfunction and ataxia in patients due to cerebellar injury.^32^, ^33^ The high proliferation of GNPs in the developing cerebellum makes them sensitive to radiotherapy,^34^ and less mature GNs are differentiated to fulfill the physiological functions of the cerebellum as a consequence. Although in early studies it had been found that the injured cerebellum could regenerate in animal models, the mechanisms have not been fully demonstrated. More detailed mechanistic studies are needed to provide strategies for the clinical development of interventions that can mitigate radiotherapy‐induced sequela. In our current study, using a radiotherapy‐induced cerebellar injury model in neonatal mice, we demonstrated the resistance and regeneration of NEPs in irradiated cerebellum and investigated the regulatory mechanisms of their regeneration.

In our previous studies, NEPs were found to be quiescent in normal status but were more tumorigenic in Shh‐medulloblastoma. Then, we found that these NEPs showed resistance to irradiation and started to proliferate after irradiation. The regeneration of NEPs in the injured cerebellum was also reported by Dr. Joyner's group, who used an in vivo mouse model to demonstrate that NEPs have the plasticity to generate GNs postinjury to recover cerebellar functions.^22^, ^35^ In our current study, we focused on the events and underlying mechanisms that contribute to NEP regeneration in the early stage of irradiation‐induced cerebellar injury. First, we found that NEPs were more resistant to irradiation both in vivo and in vitro, while they were subjected to similar DNA damage but lower DDR responses than GNPs, suggesting that quiescence and low DDR‐related gene expression probably render NEPs more tolerant to irradiation‐induced DNA damage. Then, the radioresistant NEPs switch their quiescent status to proliferative status under the regulation of the Shh signaling pathway and further differentiate into mature GNs in the irradiated cerebellum. These results were consistent with the findings of Dr. Joyner's group. Next, to investigate the triggers to initiate NEP proliferation post irradiation, we detected augmented Shh ligand at both the protein level and the mRNA level in the irradiated cerebellum, suggesting that NEPs accessed more Shh ligand in the local environment post irradiation and responded to it. In the normal developing cerebellum, Purkinje cells provide Shh ligand to support GNP proliferation.^13^, ^14^ Later, Purkinje cells were also found to provide Shh ligand to control astrocyte differentiation; thus, they play a central role in the cerebellum development.^36^ Therefore, to confirm whether the augmented Shh ligand post irradiation was provided by Purkinje cells, *Shh‐Cre‐GFP* transgenic mice were used to identify Shh‐producing cells, and the results confirmed that Purkinje cells were the main source of Shh ligand in the irradiated cerebellum. Our data indicate that not only in normal cerebellar development but also in injured conditions, Purkinje cells contribute to neural cell proliferation through Shh ligand production. Surprisingly, we found that the number of Shh‐producing Purkinje cells was increased in the irradiated cerebellum. Further measurement showed that the numbers of Purkinje cells in lobules V–X of the irradiated cerebellum were increased. Our results were inconsistent with some early studies that demonstrated that X‐irradiation affected the alignment and morphology but not the numbers of Purkinje cells in infant rats.^37^, ^38^ We observed that not only the layers in all lobules but also the numbers in lobules V–X of Purkinje cells were altered in the irradiated mouse cerebellum, which might be due to the difference in animal irradiation models. However, irradiation did not induce the proliferation of Purkinje cells. Considering that Purkinje cells are differentiated from their precursors,^39^, ^40^ we next observed the accumulation of Lhx1+ Purkinje cell precursors in the irradiated cerebellum. These results suggest that the increase in Purkinje cell numbers post irradiation resulted from their differentiation but not proliferation. Taken together, the increased Purkinje cells are Shh‐producing cells in the irradiated cerebellum; thus, the augmented Shh level should be the combined result of both increased Purkinje cell numbers and their Shh‐producing activity. Furthermore, we investigated the regulatory mechanisms of augmented Shh ligand production post irradiation. The Shh ligand is a morphogen that controls not only cerebellar development but also multiple neural cell differentiation and survival in the CNS.^12^, ^41^, ^42^, ^43^ However, the mechanism of its expression is less well understood. It was reported that IFN‐γ, an inflammatory cytokine in the immune system, could induce the expression of Shh through direct targeting of its transcription in GNPs.^25^, ^26^ Here, we observed increased IFN‐γ expression in the irradiated cerebellum, prompting us to hypothesize that it would account for the augmented Shh ligand post irradiation. To address this question, we performed in vivo administration of recombinant IFN‐γ by intracerebellum injection into naïve *Shh‐Cre‐GFP* and *Nestin‐CFP* mice and found that IFN‐γ administration increased the Shh‐producing cell number as well as NEP proliferation 48 h post administration. However, the persistence of the locally administrated IFN‐γ could not be monitored in our model, we then utilized a semi in vivo slice culture method and found that exogenous IFN‐γ administration increased Shh ligand expression in the cerebellum of naïve mice. Moreover, blocking IFN‐γ with a neutralizing antibody in the slice culture of the irradiated cerebellum counteracted the production of Shh ligand and NEP proliferation, indicating that IFN‐γ promotes NEP proliferation by upregulating Shh ligand production in the irradiation‐injured cerebellum. IFN‐γ is an important cytokine that plays critical roles in the immune defense against viral infection^44^ and inflammatory pathogenesis, including neuroinflammatory diseases.^45^, ^46^ More recent studies have demonstrated that it is also involved in neurogenesis.^47^ IFN‐γ is mainly expressed by natural killer cells and T lymphocytes, while other cells, including neural cells, are also positive for IFN‐γ expression.^48^, ^49^ Microglia, the immune cells in the brain, express IFN‐γ based upon their activation.^27^, ^28^, ^29^ Microglia can be activated by irradiation.^30^, ^31^ In our irradiation mouse model, we observed microglia but not T‐cell accumulation in the irradiated cerebellum, and microglia from the irradiated cerebellum expressed much higher levels of IFN‐ γ as well as other inflammatory cytokines, such as IL‐1α and IL‐6, than those from the controls. Certainly, other cell sources of IFN‐γ cannot be excluded in our current model, and further experiments need to be conducted to address this question. The results above revealed that in irradiation‐induced cerebellar injury, the inflammatory microenvironment favors neuron regeneration to a certain extent.

Taken together, our current study demonstrated that NEPs were radioresistant and regenerable in the developing cerebellum, and microglia were activated in the irradiated cerebellum to produce more IFN‐γ, enhancing Purkinje cell‐derived Shh ligand levels, which contributed to NEP proliferation. Mitigating the side effects of radiotherapy by transplanting stem or progenitor cells or by stimulating endogenous stem or progenitor cell proliferation has been demonstrated to improve irradiation‐induced spinal cord dysfunction in rats.^50^, ^51^ However, there are technical and ethical limitations for transplanting stem cells; thus, regulating the in situ proliferation of endogenous stem cells and progenitor cells to repair impaired brain function will become the main treatment strategy for radiotherapy‐induced brain injury. Therefore, our current study investigated the cellular and molecular mechanisms of the proliferation of a group of progenitor cells with regenerative potential, NEPs, after irradiation, which will help to provide a clinical protocol for cerebellar developmental disorders and cerebellar function recovery after radiotherapy for brain tumors.

## AUTHOR CONTRIBUTIONS

YW: conceptualization and study design, data analysis, funding acquisition, paper writing, and editing. PL: conceptualization and study design, data analysis, and paper reviewing. LZ: data analysis, funding acquisition and paper reviewing. JH, ZW, BG, and LF: experiments design and performance, data analysis and graphing. YS, SZ, LW, YQ, GL, and FD: data analysis and experiments performance. ZCN: data analysis and paper reviewing. All authors read and approved the final manuscript.

## FUNDING INFORMATION

This research was supported by National Natural Science Foundation of China (82073873 to Yuan Wang, 82072798 to Li Zhang), and Priority Academic Program Development of the Jiangsu Higher Education Institutes.

## CONFLICT OF INTEREST STATEMENT

The authors declare that there are no conflicts of interest.

## Supporting information


Figure S1:

## Data Availability

The data generated from this study are available upon request from the corresponding authors.
