# Supplementary material for: IFN‐γ promotes radioresistant Nestin‐expressing progenitor regeneration in the developing cerebellum by augmenting Shh ligand production
Source: CNS Neurosci Ther. 2023 Oct 3;30(1):e14485. doi: 10.1111/cns.14485 (PMC10805445; doi:10.1111/cns.14485)

sFigure 1 mRNA levels of Shh and Gli1 were upregulated in the irradiated cerebellum

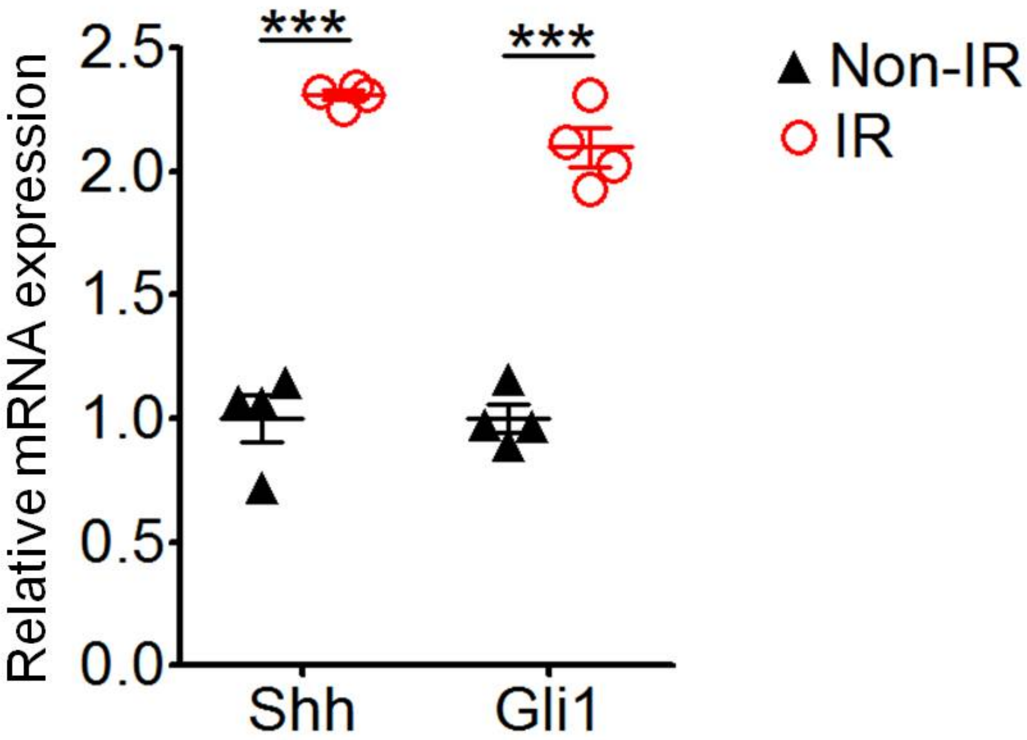

sFigure 2 NEPs responded to Shh ligand to proliferate in vitro

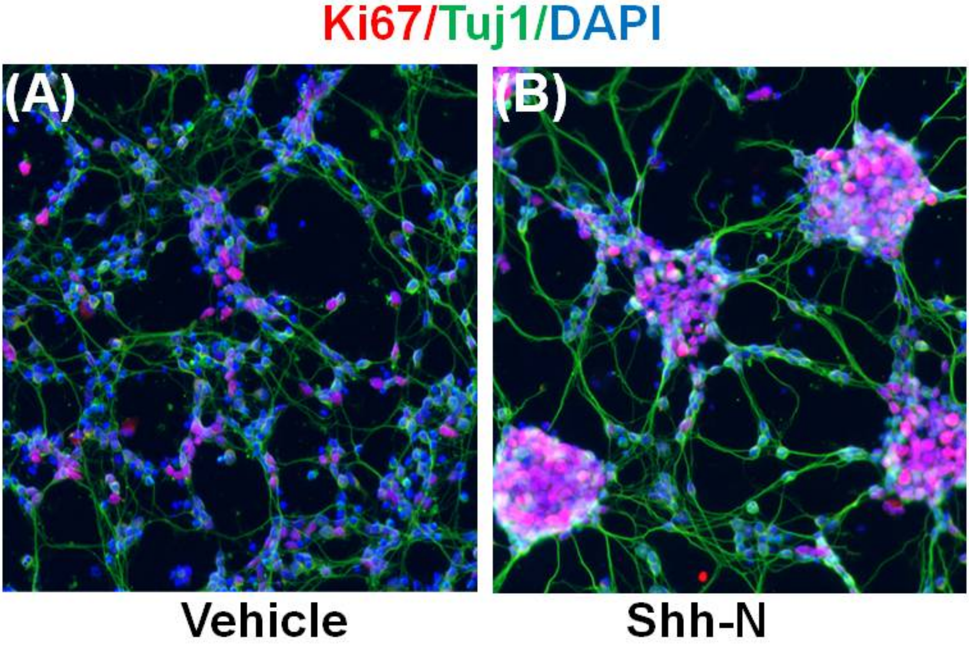

sFigure 3 The numbers of Purkinje cells and their precursors were increased in the irradiated cerebellum

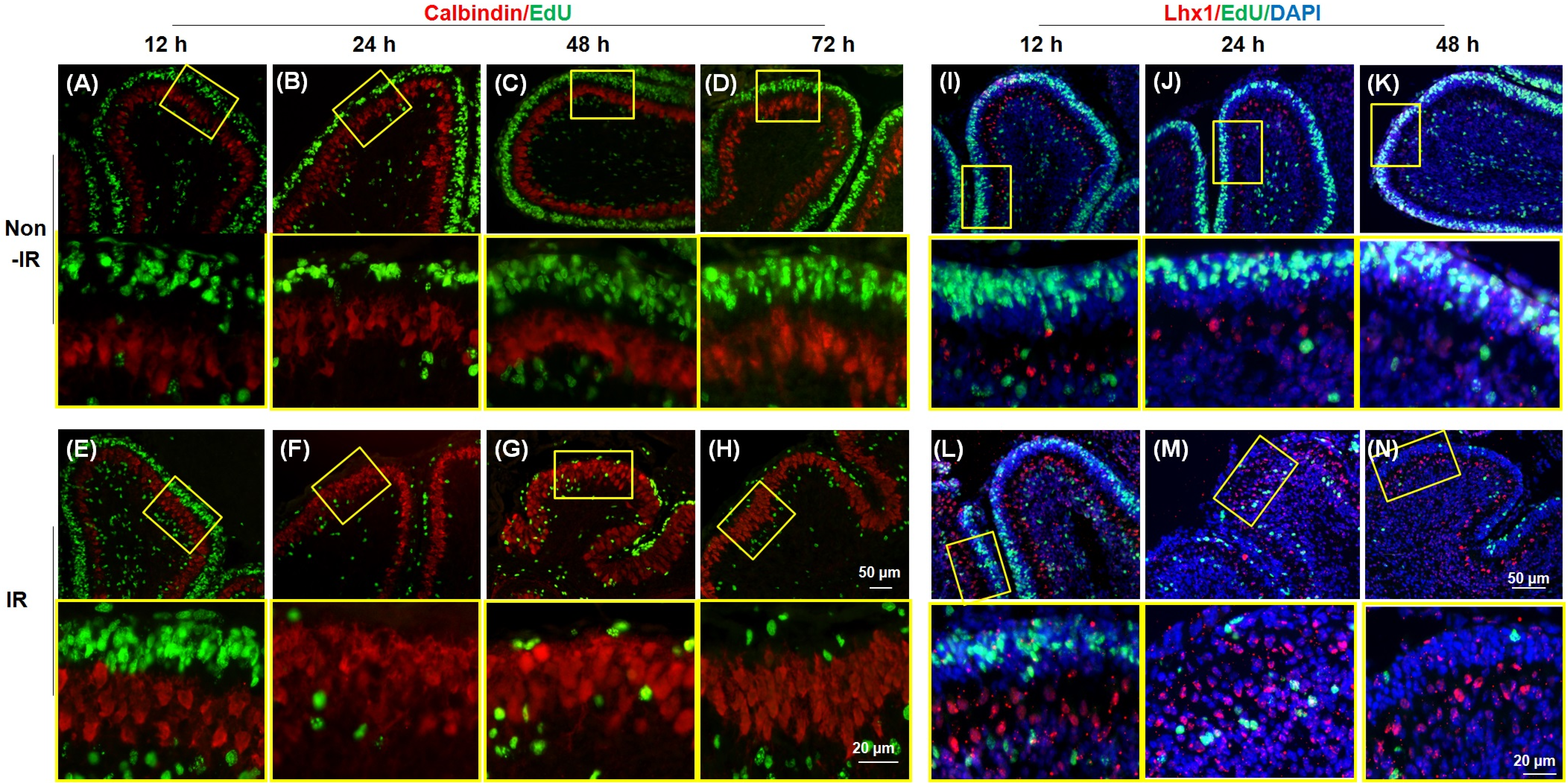

sFigure 4 INF-γ expression of T cells was comparable in the irradiated cerebellum and the non-irradiated ones

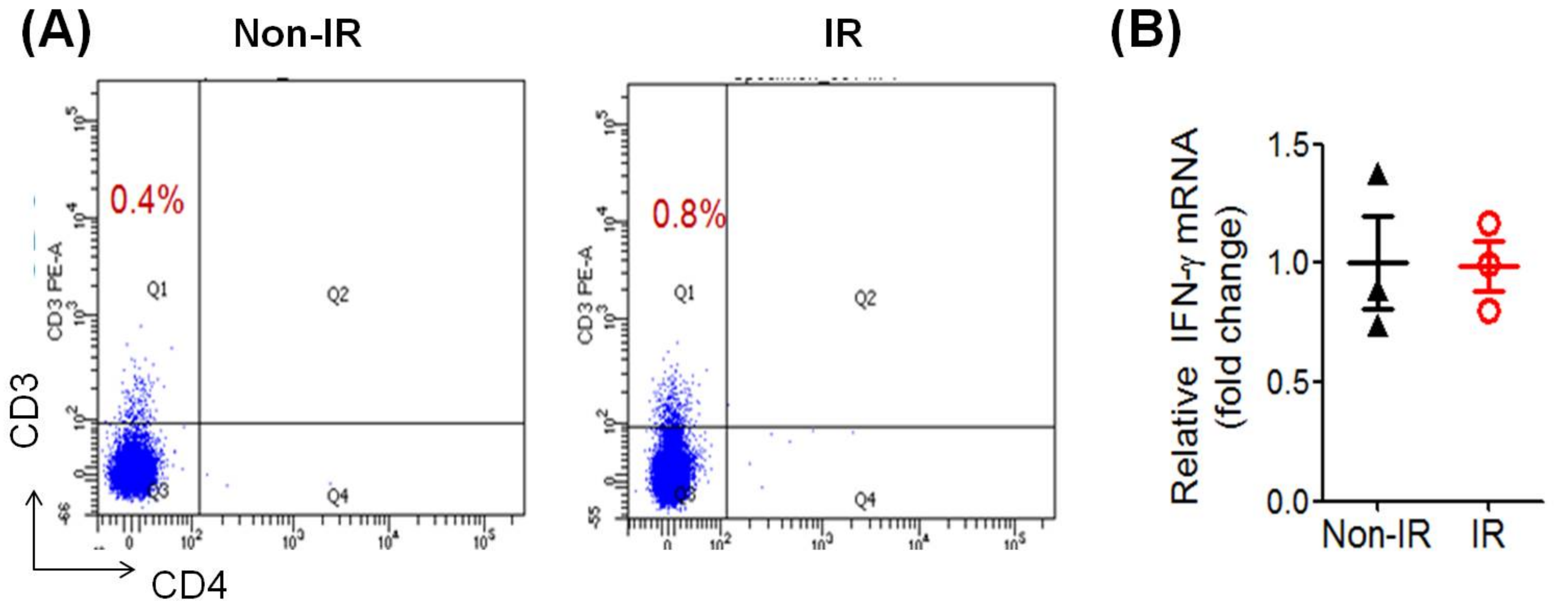

sFigure 5 IFN-γ receptors were present on Purkinje cells

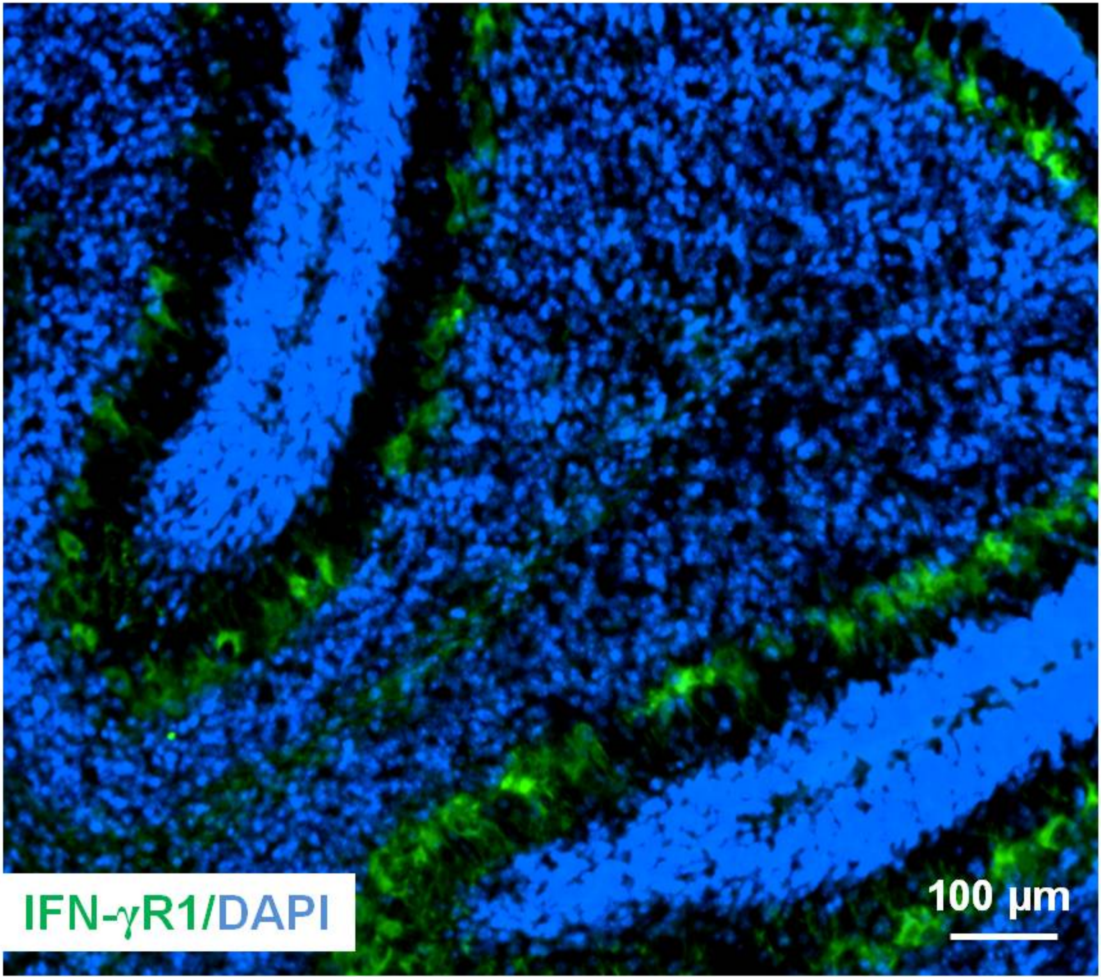

sFigure 6 mRNA levels of Shh signal pathway target genes were increased in IFN-γ treated slices

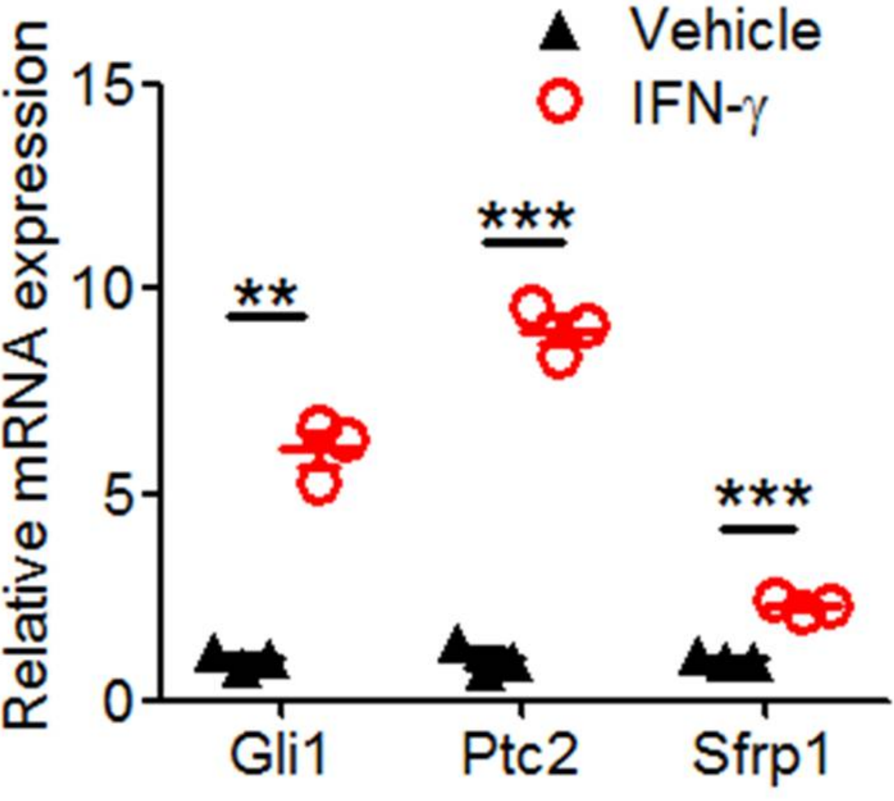

sFigure 7 Local IFN-γ injection increased the numbers of Shh+ cells and NEP proliferation

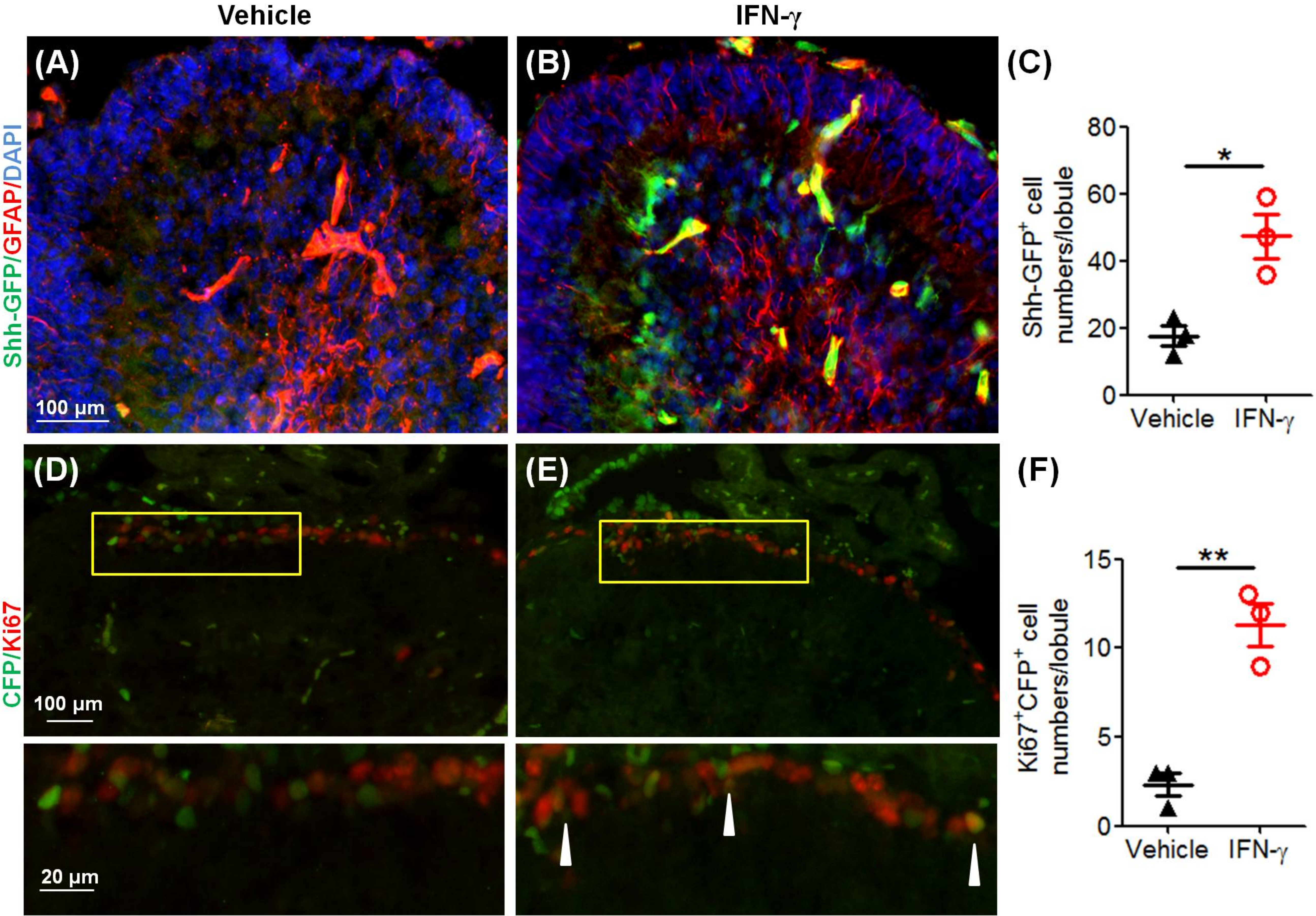

sFigure 8 NEP proliferation after irradiation in IFN-γ null mice was decreased

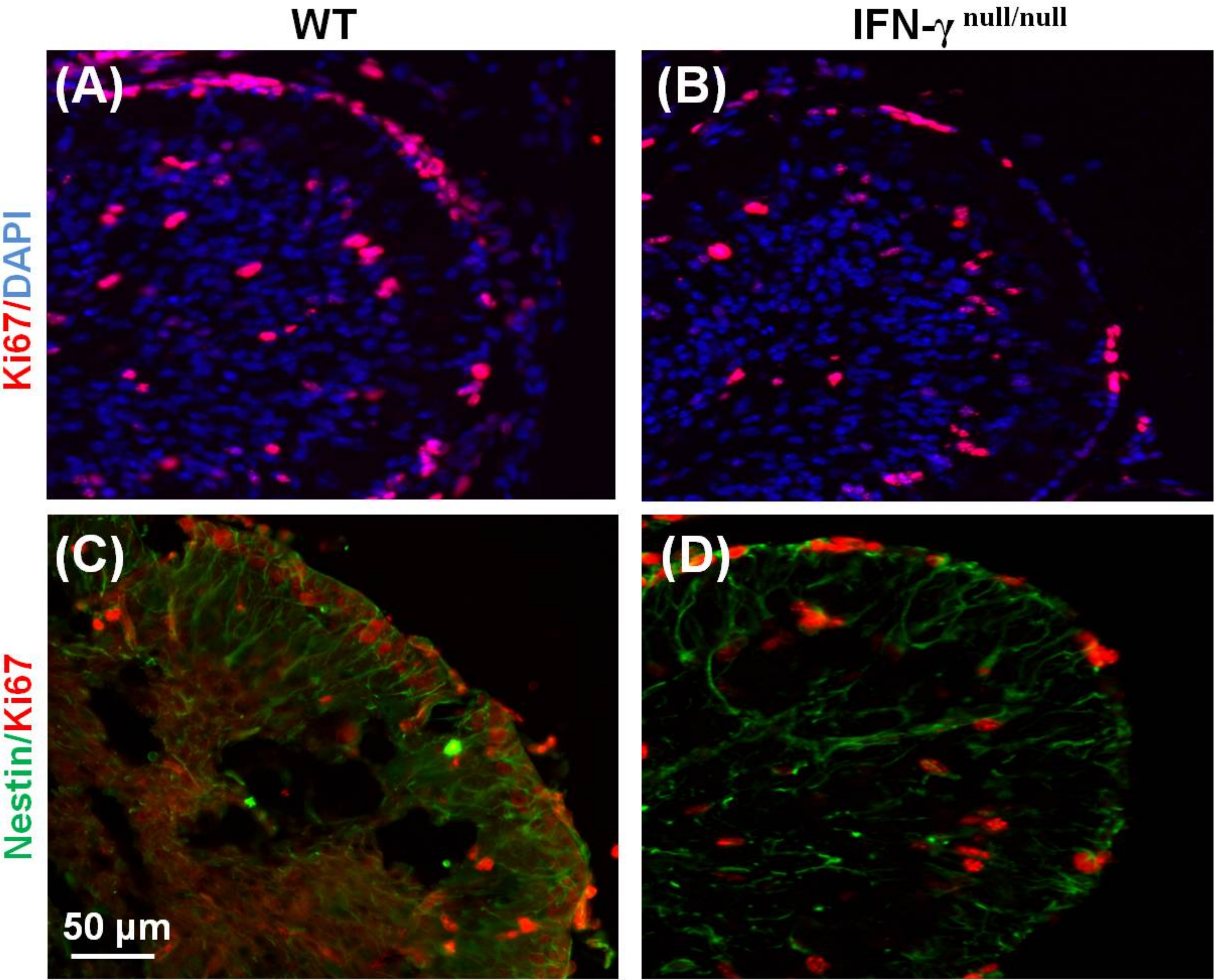

Supplement: Supplementary file 1 — Figure S1: [file CNS-30-e14485-s001.pdf]
